# Mutagenesis of the melon *Prv* gene by CRISPR/Cas9 breaks papaya ringspot virus resistance and generates an autoimmune allele with constitutive defense responses

**DOI:** 10.1093/jxb/erad156

**Published:** 2023-05-03

**Authors:** Shahar Nizan, Arie Amitzur, Tal Dahan-Meir, Jennifer I C Benichou, Amalia Bar-Ziv, Rafael Perl-Treves

**Affiliations:** The Mina and Everard Goodman Faculty of Life Sciences, Bar Ilan University, Israel; The Mina and Everard Goodman Faculty of Life Sciences, Bar Ilan University, Israel; Plant and Environmental Sciences, Weizmann Institute of Science, Israel; The Mina and Everard Goodman Faculty of Life Sciences, Bar Ilan University, Israel; The Mina and Everard Goodman Faculty of Life Sciences, Bar Ilan University, Israel; The Mina and Everard Goodman Faculty of Life Sciences, Bar Ilan University, Israel; University of Gent, Belgium

**Keywords:** CRISPR/Cas9, *Cucumis melo*, melon, papaya ring spot virus (PRSV), plant autoimmune mutation, resistance gene

## Abstract

The majority of plant disease resistance (*R*) genes encode nucleotide binding-leucine-rich repeat (NLR) proteins. In melon, two closely linked NLR genes, *Fom-1* and *Prv*, were mapped and identified as candidate genes that control resistance to *Fusarium oxysporum* f.sp. *melonis* races 0 and 2, and to papaya ringspot virus (PRSV), respectively. In this study, we validated the function of *Prv* and showed that it is essential for providing resistance against PRSV infection. We generated CRISPR/Cas9 [clustered regularly interspaced palindromic repeats (CRISPR)/CRISPR-associated protein 9] mutants using *Agrobacterium*-mediated transformation of a PRSV-resistant melon genotype, and the T_1_ progeny proved susceptible to PRSV, showing strong disease symptoms and viral spread upon infection. Three alleles having 144, 154, and ~3 kb deletions, respectively, were obtained, all of which caused loss of resistance. Interestingly, one of the *Prv* mutant alleles, *prvΔ154*, encoding a truncated product, caused an extreme dwarf phenotype, accompanied by leaf lesions, high salicylic acid levels, and defense gene expression. The autoimmune phenotype observed at 25 °C proved to be temperature dependent, being suppressed at 32 °C. This is a first report on the successful application of CRISPR/Cas9 to confirm *R* gene function in melon. Such validation opens up new opportunities for molecular breeding of disease resistance in this important vegetable crop.

## Teaser

Successful gene editing in melon proves that an *R*gene candidate, *Prv*, controls resistance to papaya ringspot virus, while one of the mutated alleles causes dwarfism and autoimmunity.

## Introduction

The plant immune system consists of two major layers of defense: the pathogen-associated molecular pattern (PAMP)-triggered immunity (PTI), and effector-triggered immunity (ETI). The first layer, PTI, is activated by plasma membrane recognition receptors (PRRs) that recognize conserved PAMP elements. The second layer, ETI, is mediated by resistance (R) proteins, that identify race-specific avirulence (AVR) factors that pathogens inject into the plant cells, eliciting a range of protective responses, such as local hypersensitive response (HR) as well as systemic defense (Jones and Dangl, 2006). Most R proteins belong to the intracellular nucleotide binding-leucine-rich repeat (NLR) family. These multidomain proteins function as a molecular switch that is turned on by the direct or indirect sensing of AVR factors, activating a defense cascade (Takken and Tameling, 2009). Their activity is tightly controlled by intra- and intermolecular interactions to prevent autoactivity in the absence of pathogens (Sukarta *et al.*, 2016). The central nucleotide-binding site (NB) domain serves as an ADP–ATP switch that regulates the ON/OFF state of the NLR protein (Bernoux *et al.*, 2016). The C-terminal leucine-rich repeat (LRR) domain, which is usually composed of repeated units with hydrophobic amino acids, acts mostly as a ligand-binding platform with autoinhibitory function (Martin *et al.*, 2003). At the N-terminus, NLR proteins carry either a coiled-coil (CC) domain, or a domain that has homology to the Toll/Interleukin-1 Receptor (TIR) domain, whose role is to transduce an activation signal to downstream defense pathways (Pan *et al.*, 2000). The recent solving of NLR protein structures greatly advanced our knowledge about their action and signaling (Ma *et al*., 2020; Martin *et al.*, 2020).

Loss of regulation of NLR proteins often leads to autoimmunity, with typical dwarf phenotypes, spontaneous HR lesion formation, and constitutive expression of defense genes (van Wersch *et al*., 2016). In Arabidopsis, mutations in TIR-NLR genes resulted in such ‘autoimmune’ phenotypes. Among these, *suppressor of npr1-1 constitutive* (*snc-1*) was studied in detail (Li *et al*., 2001). A single amino acid change in the linker region between the NB and LRR domains led to overaccumulation of the SNC1 protein and activation of salicylic acid (SA)-dependent and SA-independent defense responses (Zhang *et al.*, 2003), that contributed to enhanced pathogen resistance of the *snc-1* mutant.

*NLR* genes are often found in clusters, but a small proportion are organized in pairs, and, in a few cases, the two genes were shown to cooperate in pathogen resistance (Cesari *et al*., 2014b; Williams *et al.*, 2014; Maqbool *et al.*, 2015). In these cases, the first NLR, known as the sensor, carries an additional ‘integrated domain’, which could act as a decoy for pathogen effectors, whereas the second NLR acts as a signal transducer for activation and, in the absence of the effector, it remains inhibited by the sensor NLR (Cesari *et al*., 2014a; Williams *et al.*, 2014; Huh *et al.*, 2017).

In *Cucumis melo* L. (melon), the closely linked genes, *Fom-1* and *Prv*, confer resistance to *Fusarium oxysporum* f.sp. *melonis* (FOM) races 0 and 2, and the papaya ring spot virus (PRSV), respectively (Brotman *et al.*, 2013). PRSV is one of the limiting factors for papaya and cucurbit production worldwide (Yap *et al.*, 2009). PRSV has two pathotypes: PRSV-P infects both papaya and cucurbits, while PRSV-W infects only cucurbits (hereafter, PRSV-W will be designated PRSV). PRSV causes severe mosaic, blistering, and malformations on leaves of squash, cucumber, melon, and watermelon. Fruit may also show discoloration and deformation (Lecoq and Desbiez, 2012). Candidate genes for *Fom-1* and *Prv* were identified by positional cloning (Brotman *et al.*, 2013) using several mapping populations that segregated for PRSV and/or Fusarium resistance. They encode proteins of the TIR-NLR family and reside in a head-to-head orientation, separated by an ~1300 bp intergenic region. An unusual non-conserved domain, encoding a second NBS domain, is found in the C-terminus of *Prv*, in agreement with the ‘Integrated Decoy’ model proposed for *R* gene pairs (Cesari *et al*., 2014a). *Fom-1* and *Prv* were identified by genetic mapping, but have not been functionally validated, and their mechanism of action and possible molecular interaction have not been shown.

Genetic transformation of melon is a low efficiency, technically challenging process (Nuñez-Palenius *et al.*, 2008). Stable transformation is required for the application of gene editing tools such as CRISPR/Cas9 [clustered regularly interspaced short palindromic repeats (CRISPR)/CRISPR-associated protein 9; Hu *et al.*, 2017]. Hooghvorst *et al.* (2019) were the first to publish a preliminary CRISPR study in melon, that targeted the phytoene desaturase (PDS) gene and generated albino plantlets, but these were not grown to maturity. In 2022, a handful of papers reported melon knockout lines obtained by CRISPR/Cas9. Two of the targeted genes were involved in fruit ripening (Giordano *et al.*, 2022; Liu *et al.*, 2022), the other two affected plant–virus interactions (Leibman *et al*., 2022; Pechar *et al.*, 2022).

Here, CRISPR/Cas9 was applied for the first time to a melon *NLR* gene, and stable mutations were obtained and successfully transmitted to the T_1_ and T_2_ generations. By mutating the *Prv* candidate gene, we proved that it is essential for resistance against PRSV infection. Interestingly, in addition to the resistance-breaking effect, one of the *Prv* mutant alleles caused a severe dwarf phenotype that expressed temperature-dependent defense responses with increased SA levels.

## Materials and methods

### Plant material

Melon genotypes Charéntais Prv-R, that harbors the PRSV resistance allele *Prv*^*1*^ originating from melon WMR-29 (Pitrat and Lecoq, 1983), and the near-isogenic susceptible cultivar Charéntais-T, were kindly provided by C. Dogimont, INRA Monfavet, France. Seedlings were grown in pots containing sterilized soil in the growth room at 25 °C, under a 16:8 h light:dark photoperiod.

### CRISPR/Cas9 vector construction

Two candidate guide RNAs (gRNAs) were selected for targeting the *Prv* gene using the CRISPOR website (http://crispor.tefor.net), and version v3.5.1 of the publicly available genome sequence of melon genotype DHL92 (https://www.melonomics.net). The gRNA candidates were located upstream of an NGG protospacer adjacent motif (PAM), and selected according to several criteria: specificity score (uniqueness in the genome), predicted gRNA efficiency, and the presence of restriction enzymes at the cleavage site that can later help in screening the mutation. In addition, we looked for gRNAs with a minimum of potential off-targets sites; that is, sites located in exons that have <4 mismatches with respect to the gRNA. Both gRNAs targeted the first exon of the *Prv* gene: gRNA1, base pairs 402–421 (starting from the ATG codon), 5ʹ **CCA**TCACGTTTATCATCAAAGTC, and gRNA2, base pairs 533–552, 5ʹ ATTACTCGGAGGTCAACTAC**AGG**. The PAM site is marked in bold letters and the diagnostic restriction sites (*Tai*I or *Hin*cII, respectively) are underlined. The CRISPR/Cas9 construct was prepared using the GoldenBraid (GB) cloning system, following the assembly strategy described by Sarrion-Perdigones *et al.* (2014). GB plasmids were provided by Ms Tal Dahan and Professor Avi Levy, Weizmann Institute of Science, Israel, and cloning followed the method described by Dahan-Meir *et al.* (2018). The construct contained a kanamycin resistance gene (*npt*II) expressed under the control of the nopaline synthase (*nos*) promoter and *nos* terminator. The *Streptococcus pyogenes Cas9* gene was expressed under the constitutive tomato ubiquitin 10 promoter (Solyc07g064130, 2079 bp upstream of the ATG start codon) and the respective terminator region (1443 bp downstream of the TAA stop codon). The gRNAs were expressed under the control of the *Arabidopsis thaliana* U6-26 RNA Pol III promoter (Vazquez-Vilar *et al*., 2016). To clone the two *Prv* gRNAs, we used the following oligonucleotide pairs with GB overhang extensions required for cloning: *Prv*_gRNA1_Fw: 5ʹ-ATTG**ACTTTGATGATAAACGTGA**, *Prv*_gRNA1_Rv: 5ʹ-AAAC**TCACGTTTATCATCAAAGT**, *Prv*_gRNA2_Fw: 5ʹ-ATTG**ATTACTCGGAGGTCAACTAC**, *Prv*_gRNA2_Rv: 5ʹ-AAAC**GTAGTTGACCTCCGAGTAAT**. The gRNA target site is shown in bold letters and the GB extensions are underlined. The presence of GB plasmids in *Escherichia coli*, as well as in *Agrobacterium*, was confirmed by restriction digestion of plasmid DNA, and by Sanger sequencing.

### Checking off-target events

The CRISPOR website (http://crispor.tefor.net, Haeussler *et al.*, 2016) in combination with the public genome sequence of melon (DHL92 v3.5.1) were used to identify potential off-target sites. These were ranked according to their cutting frequency determination (CFD) score (Doench *et al.*, 2016) that incorporates criteria such as specificity score and gRNA cleavage efficiency. The primers used to amplify and sequence the off-target candidate site are given in Supplementary Table S1.

### *Agrobacterium*-mediated transformation of melon

*Agrobacterium*-mediated transformation was performed by the method of Leibman *et al*. (2011), with minor changes. Briefly, explants were prepared from the first true leaf of 2-week-old seedlings of the PRSV-resistant melon genotype, Charéntais Prv-R. *Agrobacterium tumefaciens* strain EHA105 harboring the *Prv* CRISPR/Cas9 plasmid, *Prv*-p3a1, was used. Selection of transgenic shoots was done by supplementing the media with 150 mg l^–1^ kanamycin during both regeneration and elongation stages, while 100 mg l^–1^ kanamycin was used in the rooting medium. *Agrobacterium* overgrowth was controlled by adding 500 mg l^–1^ cefotaxime. Rooted plantlets, ~5–10 cm in height, were hardened for 3 weeks in the growth room at 25 °C, under a 16:8 h light:dark photoperiod, and transplanted to the greenhouse. Melon plants of the T_0_ generation were self-pollinated to obtain T_1_ generation seeds.

### Verification of transgenic lines and detection of CRISPR/Cas9 mutations

Each regenerated plant was screened by PCR for the presence of both the *nptII* and *Cas9* transgenes using the primers listed in Supplementary Table S1. Plant DNA extraction was according to Dellaporta e*t al.* (1983) and ~100 ng of DNA was used as template. To detect and characterize Cas9-induced mutations, the target region in the *Prv* gene was PCR-amplified using primers that encompass both targets, yielding a 607 bp product. PCR was carried out by Phusion™ High-Fidelity DNA Polymerase (Thermo Fisher Scientific, USA), according to the manufacturer’s instructions. PCR products of transformed, as well as wild-type (WT) plants, were digested with FastDigest restriction endonucleases *Hin*cII and *Tai*I (Thermo Fisher Scientific, USA) that cut upstream of the PAM motif. Restriction products were separated on agarose gels, to see whether restriction sites have been abolished. To further characterize the mutated alleles, PCR products were purified using the Gel/PCR DNA Fragments Kit (Geneaid, Taiwan) and sequenced. In bi-allelic situations, where the allelic mixture could not be sequenced directly, products were cloned into the pGEM-T easy plasmid (Promega) following standard protocols. Plasmid DNA from 5–6 colonies from each ligation was extracted by the Presto™ Mini Plasmid Kit (Geneaid, Taiwan) following the manufacturer’s instructions, and sequenced.

### Inoculation of plants with PRSV

Two-week-old melon seedlings of the T_1_ and T_2_ generations were inoculated with PRSV using tungsten particles coated with DNA of a PRSV-E2 infective plasmid (Desbiez *et al.*, 2012) and a biolistic device (Gaba *et al.*, 2013). In a few experiments, we used a PRSV-green fluorescent protein (GFP) clone in which we inserted a multiple cloning site with an NIa protease site in the PRSV-E2 clone, between the P1 and HC-Pro coding sequences, and then cloned the mGFP reading frame, delimited by protease recognition sequences, resulting in a GFP-expressing virus having an infectivity similar to that of the parent E2 clone. The two cotyledons of each seedling were bombarded, and PRSV symptoms were observed 2–3 weeks post-inoculation. Three biological replicates were performed, consisting of separate experiments carried out on different days. In each experiment, 10 plants from each genotype were inoculated, and additional non-inoculated seedlings (10 per genotype) were left as a control. Proportions of survival were compared between different genotypes using the Cochran–Mantel–Haenszel test. Post-hoc analysis was performed by repeating the test for each pair of treatments followed by Benjamini–Hochberg [false discovery rate (FDR)] adjustment of *P*-values for multiple comparisons.

### RNA extraction, cDNA preparation, sqRT–PCR, and qRT-PCR

Total RNA was extracted from 100 mg of frozen melon leaf tissue using RiboEX (Geneall biotechnology, Seoul, Korea), according to the manufacturer’s instructions. First-strand cDNA was synthesized with a mixture of random and oligo(dT) primers, using the qScript Flex cDNA Kit (QuantaBio, USA). For semi-quantitative reverse transcription–PCR (sqRT–PCR), diluted cDNA samples containing ~5 ng and 0.5 µM of each primer were used. The number of PCR cycles was optimized for each gene, to allow visual comparison of amplicon band intensity on agarose gels. Results were normalized against the housekeeping gene, *ADP ribosylation factor 1* (*ADP*, MELO3C023630). For quantitative real-time PCR (qRT-PCR), we used the automated BioMark™ HD real-time PCR System with the GE 48.48 Dynamic Array™ (Fluidigm), at the Life Sciences Core Facilities unit of Weizmann Institute of Science. Samples contained 50 ng μl^–1^ cDNA and 100 µM primers, and the results were analyzed using the Fluidigm Real-Time Analysis Software (Fluidigm). Four melon housekeeping genes were tested, namely *ADP* (MELO3C023630), *cytosolic ribosomal protein S15* (*CmRPS15*, MELO3C006471), *Ubiquitin-conjugating enzyme E2* (*Ubi*, MELO3C019589), and *ribosomal protein L2* (*L2*, MELO3C000111; Kong *et al.*, 2014, 2016). Of these, *ADP* was used as the reference gene to normalize the qRT-PCR data. The list of genes and primers is provided in Supplementary Table S1. For each gene, 3–4 independent biological repeats were analyzed, with each replicate prepared from a single plant. For each sample, the relative expression level was determined by first normalizing the Ct value against the Ct value of the *ADP* gene, generating ΔCt values. ΔΔCt values were calculated relative to the average values of the WT samples grown at 25 °C. The relative quantity (RQ) of transcripts was calculated by the Livak–Schmittgen method, where RQ=2^−ΔΔCt^. To determine significant (*P*<0.05) differences between genotypes and temperature regimes, we applied two-way ANOVA, followed by Tukey’s post-hoc analysis.

### Trypan blue staining

Trypan blue staining was applied to visualize cell death according to Fernández-Bautista *et al.* (2016), using leaf discs of 1 cm diameter taken from 1-month-old plants. Stained tissue was cleared in 100% ethanol overnight at room temperature and visualized under a Leica Stereomicroscope M205.

### Determination of phytohormones

Phytohormones were extracted and assayed at the Life Sciences Core Facilities of the Weizmann Institute of Science, Israel by Dr Maxim Itkin. Extraction of phytohormones was carried out based on the methods described by Breitel *et al.* (2016), Dobrev and Vankova (2012), and Kojima *et al.* (2009). Finely ground, frozen plant tissue samples of 65–100 mg were extracted overnight at –20 °C with 1 ml of methanol/water/formic acid (15/4/1 v/v/v), containing 25 µl of 1 µg ml^–1^ d4-SA and 0.1 µg ml^–1^ d6-abscisic acid (ABA) (OlChemim, Czech Republic) as stable isotope-labeled internal standards, followed by a second extraction with 0.5 ml of the same solvent without internal standards. After evaporation under a gentle nitrogen stream and reconstitution to 1 ml of 1% acetic acid in double-distilled water (DDW), the extract was applied on an Oasis MCX 1 ml/30 ml cartridge pre-conditioned with 1 ml of acetonitrile (ACN), 1 ml of MeOH, 0.5 ml of 0.1 M HCl, and 1 ml of 1% acetic acid. Then, the cartridges were washed with 1 ml of 1% acetic acid in DDW, eluted with 1.5 ml of MeOH, and eluents were evaporated to dryness under a gentle nitrogen stream and reconstituted in 50 µl of 5% ACN in DDW. Phytohormones were measured using the UPLC ACQUITY (Waters Corp., MA, USA) system coupled to a TQ-XS mass spectrometer (Waters Corp.). The chromatographic separation was performed on an ACQUITY UPLC BEH C18 column (2.1 × 100 mm, i.d., 1.7 μm, Waters Corp.). Mobile phase A consisted of 3% ACN in DDW when mobile phase B was 100% ACN; both were supplemented with 0.1% acetic acid. The column was maintained at 35 °C and the flow rate of the mobile phase was 0.3 ml min^–1^. Initially, 5% B was run for 1 min, followed by a 1 min gradient to 15% B and a 10.5 min gradient to 45% B. This was followed by a 1 min run at 45% B, 0.5 min gradient to 100% B, and 1.5 min wash with 100% B. After that, the system was set to the initial conditions in 0.25 min, and equilibrated for 1.25 min. The measurement was performed in multiple reaction monitoring (MRM) mode, using two MRM traces for each compound. Capillary voltage was 2.5 kV. The phytohormones that were measured and additional MS parameters are summarized in Supplementary Table S2. Data were processed with MassLynx software with Targetlynx (Waters). Quantification of SA and ABA was done against external calibration curves, using the analyte/internal standard peak ratio. Jasmonic acid (JA) was only relatively quantified by peak area.

## Results

### Generation of *Prv* knockout lines using CRISPR/Cas9

**Fig. 1.**
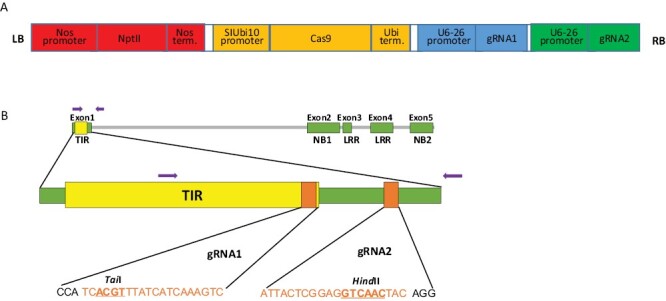
Gene editing of the *Prv* gene by CRISPR/Cas9. (A) Schematic representation of the CRISPR/Cas9 expression cassette containing the *Streptococcus pyogenes* Cas9 nuclease under the constitutive *Solanum lycopersicum* ubiquitin10 promoter (SIUbi10) (Solyc07g064130, 2079 bp upstream of the start codon), and the respective terminator region (1443 bp downstream of the stop codon). Each of the two gRNAs was expressed under the control of the *Arabidopsis thaliana* U6-26 RNA Pol III promoter, and the *NptII* selective marker was expressed under the nopaline synthase (nos) promoter and nos terminator. (B) Schematic representation of the melon *Prv* locus and the gRNA target sites (orange boxes). The gRNA target sequences are shown in orange letters, the restriction site near the target is shown in bold, and the protospacer adjacent motif (PAM) is in black. The purple arrows indicate the primers flanking the target sites, that were used to detect the mutations.

In our previous studies, the melon *Prv* resistance gene, MELO3C022145, was located by positional cloning on melon chromosome 9, closely linked to the *Fom-1* gene, MELO3C022146 (Brotman *et al.,* 2013). In order to validate its function in conferring resistance to PRSV, we set out to inactivate it using the CRISPR/Cas9 system. We applied *Agrobacterium*-mediated transformation to the PRSV-resistant melon genotype Charéntais Prv-R, whose resistance had been derived by sexual crosses from melon breeding line WMR-29 (Pitrat and Lecoq, 1983). Using the GB 2.0 cloning method, we generated a binary plasmid that harbored, within the T-DNA region, *Streptococcus pyogenes* Cas9 nuclease under the *Solanum lycopersicum* Ubiquitin promoter, and the *NptII* selective marker for kanamycin resistance (Fig. 1A). We cloned in tandem, in the same T-DNA region, two gRNA genes that targeted two sites in the first exon of *Prv*, under the U6-26 promoter (Dahan-Meir *et al.*, 2018) (Fig. 1B).

**Fig. 2.**
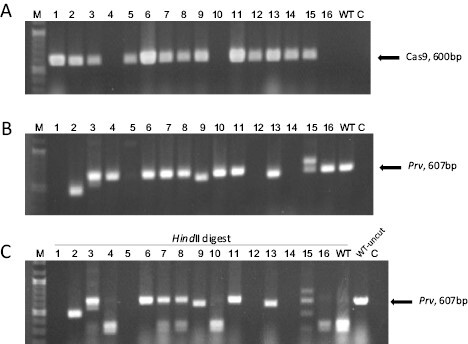
Genotyping of T_0_ generation plants by PCR. Samples 1–16 indicate independent regenerated plants. (A) PCR analysis for the presence of *SpCas9*, used to confirm the transgenic plants. (B) PCR analysis of the *Prv* target region (607 bp in the WT), to detect mutations in the T_0_ plants. (C) Restriction digestion of the *Prv* amplicons from (B) with *Hin*dII. On the right, an uncut WT amplicon is shown for comparison. Undigested amplicons indicate mutations in both alleles, while partially digested amplicons are probably due to heterozygosity. C, negative control without template DNA; M, molecular weight marker.

Starting from leaf explants, shoots were regenerated and rooted on kanamycin-selective medium. Sixteen T_0_ generation plants were obtained and tested for transgene presence by PCR, using *Cas9*-specific primers (Fig. 2A). Thirteen plants were confirmed as independent transgenic plants, while Plants 4, 10, and 16 were Cas9 negative and represented non-transgenic ‘escapes’ that survived kanamycin selection. To test the presence of mutations in the target gene, PCR was applied to the same samples using primers that flanked the two gRNA target sites in the *Prv* gene, producing a 607 bp amplicon in the WT (Fig. 2B). Interestingly, electrophoresis of the PCR products readily revealed several small visible deletions (>50 bp, samples 2, 9, and 13) as well as larger ones that resulted in no amplification of the target, suggesting that the deletion encompassed annealing sites of one or both primers (samples 1, 5, 12, and 14). Moreover, these plants had deletions in both alleles, since no WT size amplicon was present. Other plants had amplicons that appeared similar in size to the WT amplicon. To facilitate the diagnosis of mutated alleles, the two gRNA target sites were designed in proximity to restriction enzyme recognition sites (*Hin*dII and *Tai*I, respectively) such that a mutation 3 bp upstream of the PAM site will have a high probability of abolishing the restriction site (Jinek *et al.*, 2012). The product of restriction of the same amplicons with *Hin*dII is shown in Fig. 2C. The WT amplicon from non-transformed plants (as well as the alleles from the Cas9-negative plants) were fully digested, whereas Plants 3, 6, and 11 show non-digested amplicons, indicative of mutations in the target site that altered the restriction enzyme recognition sequence on both alleles. Plants 7 and 8, on the other hand, were heterozygous, showing digested as well as undigested products. Plant 15 had a visible insertion in one allele (confirmed by sequencing, below) as well as an allele whose size was similar to that of the WT.

To further characterize the mutated alleles, we cloned the PCR products encompassing the gRNA target region from individual T_0_ plants (except for Plants 1, 5, 12, and 14, where the alleles had larger deletions that prevented primer annealing). Plasmids from six bacterial colonies of each individual were sequenced. The Cas9-negative plants (nos 4, 10, and 16) had WT alleles, as expected, while Plants 7 and 8, that had been diagnosed as heterozygotes based on restriction analysis (Fig. 2C), gave rise to both WT and mutated sequences. The remaining 11 Cas9-positive plants had mutations in both alleles, and the PCR products represented a mixture of the two mutant alleles (e.g. Plants 2, 9, and 13). In total, out of 13 transgenic plants, we detected 24 mutated and two WT alleles (in Plants 7 and 8), representing a mutation efficiency of 92%. Table 1 summarizes the molecular information obtained on the alleles of the 13 transgenic plants based on both electrophoresis and sequencing. Most of the sequenced mutations involved the gRNA2 target, and contained two insertions (one of 111 bp and a single base pair insertion), and 15 deletions ranging from 5 bp to 154 bp.

**Table 1.**
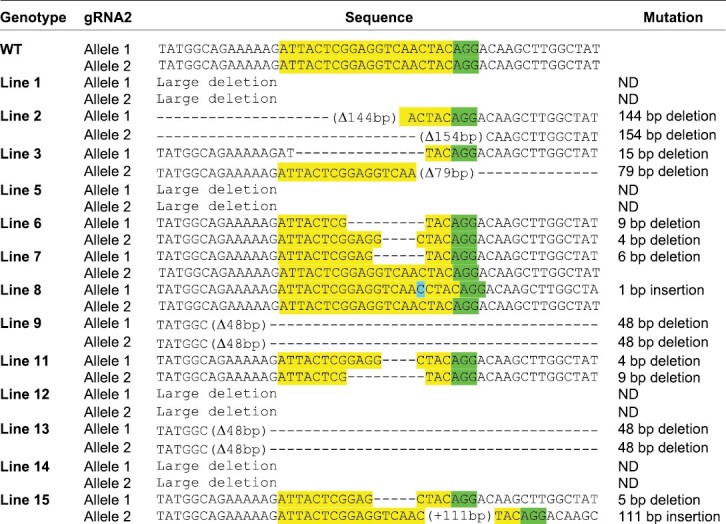
Mutations found in individual T_0_ plants

The *Prv* region encompassing the two gRNA targets was amplified, and amplicons were cloned into pGEM-T-easy vector (Promega). Six colonies of each cloning reaction were sequenced. Nine plants produced PCR amplicons, whereas four plants had deletions that abolished primer annealing and were not sequenced. The sequence of the gRNA2 target is shown in yellow and green (PAM), compared with the WT sequence. All alleles had mutations in the gRNA2 target, and the larger deletions affected both targets.

While growing the T_0_ generation plants, we noted that 11 of the 13 plants exhibited morphological abnormalities, typical of tetraploid melons, which were described in detail by Nuñez-Palenius *et al.* (2008): thick and shorter internodes, larger flowers with frequent irregular petal numbers, square-shape tetra-porous pollen grains, and small fruit with a large blossom scar. Seeds were rounder and had a thicker coat (Supplementary Fig. S1). These plants were near-sterile and could not be subjected to progeny testing for their PRSV resistance phenotype. The remaining two transgenic plants (numbers 1 and 2) looked diploid and were fully fertile. Upon self-pollination, transgenic T_1_ families 1 and 2 were obtained, that served for the subsequent experiments described below.

**Fig. 3.**
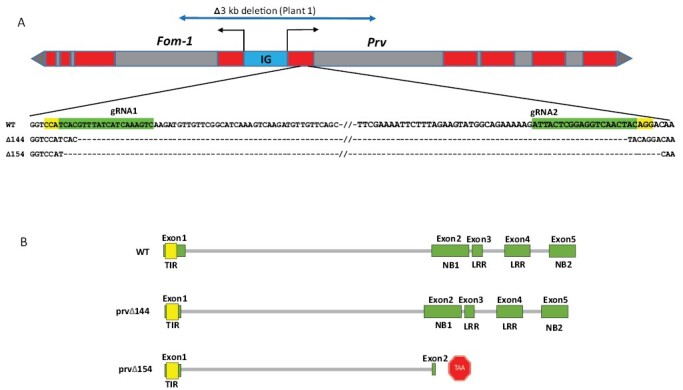
Mutations found in the *Prv* locus in T_0_ generation Plant 1 and Plant 2. (A) Map of the *Prv* locus with the mutated alleles. The *Prv–Fom-1* gene pair is depicted with exons in red, and the region between the two start codons (IG) in blue. Plant 1 has a large deletion of ~3 kb in both alleles, drawn on top of the locus, ranging from the first intron of *Fom-1* to the first intron of *Prv*. Plant 2 has smaller deletions in both alleles, spanning 154 bp and 144 bp, respectively, between the two gRNA targets. The region comprising the two CRISPR targets in the first exon of *Prv* is enlarged; the targets are emphasized in green with the PAM site in yellow. (B) Predicted translation products of WT *Prv*, compared with the two mutated alleles of Plant 2, confirmed by cDNA isolation and sequencing. Mutation Δ144 causes a 48 amino acid deletion in exon 1 but the remainder of the ORF is not affected, whereas mutation Δ154 results in a deletion in exon 1 followed by a frameshift and truncation of the translation product.

Transgenic Plant 1 had a large deletion (~3 kb) in both alleles. The deletion destroyed the coding sequences of both *Prv* and *Fom-1*. It encompassed the two target sites in *Prv* exon 1, the entire intergenic region between *Prv* and *Fom-1*, and the first exon of the adjacent gene, *Fom-1*, while the second exons of both genes and part of their first introns were still present (Fig. 3).

Plant 2 carried deletions in both alleles between the two gRNA targets, measuring 144 bp and 154 bp, respectively (Fig. 3A). As a result, the predicted TIR region of the Δ144 allele lacks 48 amino acids in the terminal part of the TIR domain, while the Δ154 deletion results in a frameshift at position 138, and a premature stop codon after another 32 missense amino acids, resulting in a truncated protein of 167 amino acids. Isolation of leaf RNA and sequencing of derived cDNA molecules from the WT and the two deletion alleles confirmed the gene models shown in Fig. 3B, and proved that the first intron in the two mutant allele transcripts is correctly spliced. The sequences of the cDNAs and their predicted translation products are provided in Supplementary Fig. S2.

To test the genetic transmission of the CRISPR-induced mutations, the T_1_ progeny of T_0_ Plants 1 and 2 were analyzed by PCR followed by gel electrophoresis. We found that 10 out of 10 T_1_ individuals from both T_1_ families had the deleted alleles and no WT size alleles, confirming that the T_0_ plants had been mutated in both alleles.

We wished to exclude the possibility that Plants 1 and 2 carry additional mutations in potential off-targets in the genome. The only likely off-target predicted by the CRISPOR software resembled gRNA2 and resided in a phosphoglycerate kinase gene, MELO3C004339. PCR amplicons of this region (primers given in Supplementary Table S1) were sequenced from WT Charéntais Prv-R, and representative T_0_, T_1_, and T_2_ plants, respectively. The sequences were identical to those of the WT in all samples.

### A dwarf mutation segregates in the T_1_ progeny of line 2

**Fig. 4.**
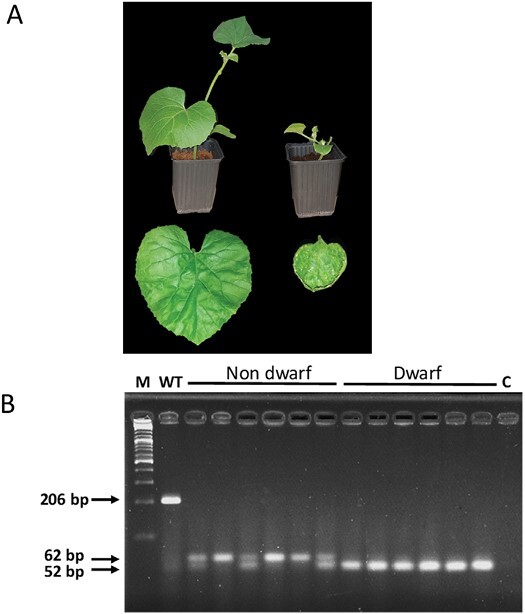
Dwarf phenotype segregating in the T_1_ progeny of Plant 2. (A) Left: normal-looking plant, with an enlarged view of a mature leaf (leaf length=7 cm). Right: dwarf T_1_ individual of the same age, with an enlarged view of a mature leaf (length=3 cm) showing spontaneous lesions. (B) PCR analysis of the *Prv* target region in a sample of 12 T_1_ progeny derived by self-fertilization of Plant 2, compared with the WT. All T_1_ plants carry deleted *Prv* alleles. The WT amplicon was 206 bp, whereas the two mutated alleles yielded 62 bp and 52 bp amplicons, respectively, separated on a 3.5% agarose TBE gel. Gel electrophoresis (confirmed by sequencing) shows that all dwarf individuals are homozygous for allele Δ154, whereas non-dwarf mutants are either homozygous for the slower allele, Δ144, or heterozygous for both alleles.

When we first grew the T_1_ generation in the growth chamber, we noticed that part of the T_1_ progeny of Plant 2 developed severe dwarfism, with short stems and very small leaves bearing small spontaneous lesions. The majority of the progeny were unaffected and had a normal phenotype (Fig. 4). Out of 70 T_1_ seedlings from self-fertilized Plant 2, 18/70 (25.7%) showed the dwarf phenotype, corresponding to 3:1 segregation of a single recessive gene (Table 2).

**Table 2.** Inheritance of the dwarf phenotype and *Prv* mutated alleles

Generation	Cross	Total no. of plants	No. of dwarf phenotype	No. of ‘normal’ phenotype	d.f.	χ^2^	Goodness of fit
T_1_	Selfed T_0_ plant No. 2	70	18	52	1	0.019	0.89
T_2_	Selfed bi-allelic T_1_ individual	24	6 (Δ154 homozygous)	18 (11 biallelic: 7 Δ144 homozygous)	2	0.25	0.88
T_2_	Selfed dwarf T_1_ individual	24	24 (all Δ154 homozygous)	0			
T_2_	Selfed Δ144 homozygous T_1_ individual	8	0	8 (all Δ144 homozygous)			

Samples of T_1_ generation progeny, and the T_2_ progeny of T_1_ individuals of the three possible genotypes, were observed for plant phenotype and tested by PCR and sequencing of the *Prv* allele (genotype given in parentheses). Where two categories were present, a χ ^2^ test was applied for the expected 3:1 gene segregation.

We asked whether the difference between the two phenotypic classes in Plant 2 progeny was related to the allelic composition in the *Prv* gene. We amplified by PCR the *Prv* target site in 12 T_1_ seedlings, six having a normal phenotype and six dwarf, and saw that all 12 had biallelic deletions in the amplicon (Fig. 4B). The amplicons were sequenced and all the dwarf individuals were found to be homozygous for the 154 bp deletion (Δ154/Δ154), while the normal-stature individuals were either homozygous for the 144 bp deletion (Δ144/ Δ144) or bi-allelic (Δ154/Δ144), associating the recessive dwarf phenotype with the Δ154 allele. To confirm such a model, T_2_ progeny of individual T_1_ self-fertilized plants were phenotypically analyzed. In the T_2_ generation, all the progeny of homozygous non-dwarf Δ144/ Δ144 plants (eight of eight tested) had normal stature, and all the progeny of dwarf plants were dwarf (24 of 24), while the T_2_ progeny of biallelic individuals (Δ154/Δ144) segregated three normal:one dwarf, and genotyping confirmed the expected segregation of the alleles (Table 2). Together, these data confirmed that dwarfism is not the result of an independent mutation, and is specified by the Δ154 allele of *Prv*. In fact, mutations in NLR genes were often reported to produce dwarf, autoimmune phenotypes (see below).

### Mutations in *Prv* break PRSV resistance

**Fig. 5.**
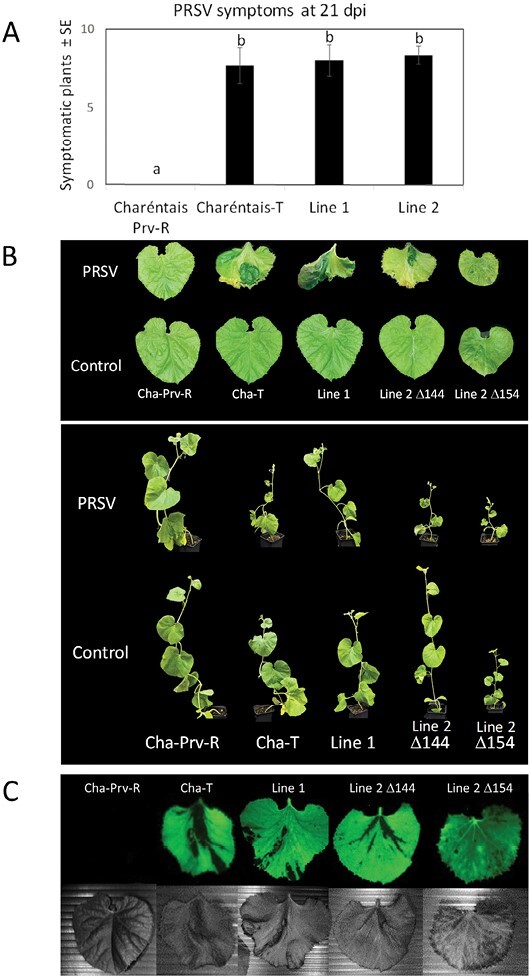
*Prv* mutants generated in a PRSV-resistant background became susceptible to PRSV infection. (A) Proportion of diseased seedlings 20 d after PRSV infection of the PRSV-resistant genotype Charéntais -Prv-R, susceptible Charéntais-T, and the T_1_ progeny of *prv* mutants from families 1 and 2. Averages are from three biological replicates, 10 seedlings per treatment×genotype combination. Proportions of survival were compared using the Cochran–Mantel–Haenszel test, followed by post-hoc analysis, with Benjamini–Hochberg (FDR) adjustment of *P*-values for multiple comparisons. (B) Representative plants and leaves of the above genotypes at 20 dpi, compared with the non-inoculated control. For family 2 progeny, a homozygous mutant for the *Δ144* allele, and a homozygous one for the *Δ154* allele displaying dwarf phenotype, are shown. (C) Expression of GFP in systemic leaves of the above genotypes, 20 dpi with a PRSV:mGFP4 strain. Leaves were observed under the Maestro II *in vivo* imaging system, 2D planar fluorescence imaging of small animals (Cambridge Research and Instrumentation, Woburn, MA, USA), using a blue excitation/emission filter set (λex, 434–480 nm; λem, >500 nm). Fluorescence intensity values were calculated as the average intensity over the leaf surface area by the Cri Maestro software. Top row, GFP fluorescence; bottom, white light image.

Mutant Plants 1 and 2 were produced by transforming Charéntais-Prv-R, a PRSV-resistant genotype, homozygous for the dominant *Prv* allele. To validate *Prv* function, we tested whether the CRISPR/Cas9-induced mutations in the gene broke PRSV resistance. T_1_ seedlings from both families 1 and 2 were inoculated with PRSV strain E2, using tungsten particles coated with DNA of a PRSV infective clone. The experiment was repeated three times with similar results, and Fig. 5A depicts the average susceptibility rates of the three replicates. At 14–21 days post-inoculation (dpi), a large proportion (~80%) of the bombarded T_1_ seedlings from both families showed severe mosaic symptoms and growth retardation. These represent typical rates of successful infection in such experiments, similar to those of susceptible Charéntais-T plants, suggesting that both alleles of both Plant 1 and Plant 2 have been functionally inactivated. In contrast, WT Charéntais Prv-R plants did not display any disease symptoms, and looked like non-infected plants. This confirms the role of *Prv* as the gene responsible for PRSV resistance in Charéntais-Prv-R. Figure 5B shows representative plants and leaves following PRSV inoculation, compared with the non-inoculated control. For family 2, that segregated for two mutated alleles, Fig. 5B shows a representative plant of each homozygous class. When T_1_ generation plants that were homozygous for each of the mutations were crossed with the resistant genotype, Charéntais-Prv-R, the resulting F_1_ progeny were resistant (10 out of 10 plants assayed), confirming that the mutated, resistance-breaking alleles are recessive with respect to the original *Prv* WT allele.

Because the severe dwarf phenotype exhibited by a quarter of the progeny of family 2 interfered with visual identification of viral symptoms, we inoculated additional T_1_ samples of both families 1 and 2 with a PRSV:mGFP4 infective clone that we generated. The recombinant isolate expresses the fluorescent protein GFP as part of the virus polyprotein, between two proteolytic cleavage sites, and it proved to be as virulent as the native E2 isolate: both mutant T_1_ families exhibited mosaic symptoms, and systemic leaves from the different genotypes were examined 14–21 dpi under a fluorescence stereo-microscope, as well as an *in vivo* imaging device. Mutant seedlings of both families showed GFP expression that correlated with disease symptoms, as did the susceptible WT plants, whereas WT resistant plants did not display any fluorescence, or disease symptoms, showing the efficacy of the *Prv* gene in restricting viral spread (Fig. 5C). Virus spread was also observed in the dwarf progeny of family 2, confirming the susceptibility of both dwarf and non-dwarf T_1_ individuals to PRSV.

### Dwarf *prvΔ154* plants show spontaneous cell death and elevated defense gene expression

**Fig. 6.**
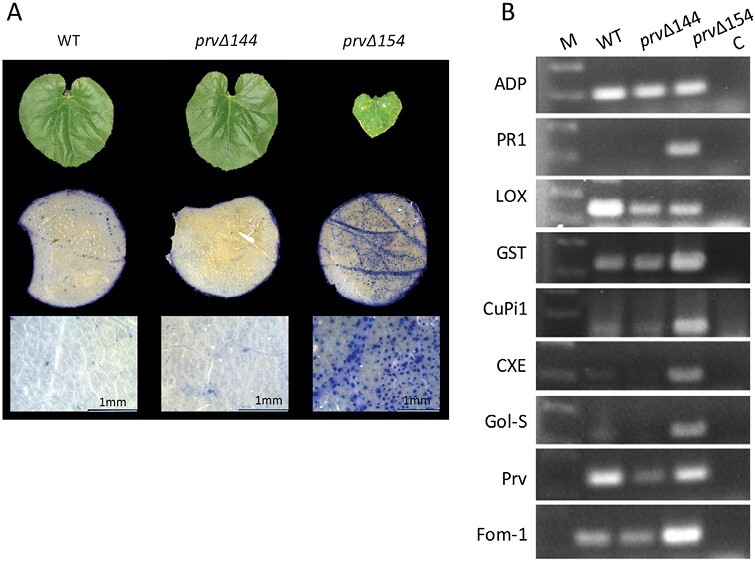
Spontaneous cell death and elevated defense gene expression in *prvΔ154* plants. (A) Trypan blue staining in *prvΔ154*, *prvΔ144*, and WT leaves. (B) sqRT–PCR assay of defense-related genes in *prvΔ154*, *prvΔ144*, and WT plants. *ADP* served as the reference gene; C, control PCR without template DNA; M, molecular weight marker. Gene identification is shown in Supplementary Table S1.

We asked whether the dwarf phenotype associated with the *prvΔ154* mutation results from an autoimmune response (i.e. constitutive overactivation of plant defense; van Wersch *et al*., 2016). To visualize cell death, leaves of *prvΔ154* and *prvΔ144* homozygous mutants and WT plants were stained with trypan blue. The leaves of the dwarf mutant were densely covered with stained cell clusters, particularly near the veins, suggesting that they underwent cell death, whereas WT and *prvΔ144* leaves had little stain (Fig. 6A).

Next, we applied sqRT–PCR to analyze the expression of the mutated gene, *prv*, its neighbor *Fom-1*, and a few defense-related gene markers that represented the SA and JA defense pathways. Defense genes were selected based on the literature, or based on their marked induction by pathogens in RNAseq experiments found in the Cucurbit Genomic website http://cucurbitgenomics.org/rnaseq/home. Gene number reference and the primer pairs used are listed in Supplementary Table S1.

Total RNA and cDNA samples were prepared from young leaves of homozygous *prvΔ154* mutant dwarf plants, homozygous *prvΔ144* (non-dwarf mutants), and WT Charentais-Prv-R plants, grown in the absence of pathogens at 25 °C in the growth chamber. The number of PCR cycles was optimized to allow visual estimation of the amplicon abundance on agarose gels. Figure 6B shows the sqRT–PCR products of a single biological replicate, out of three replicates taken from different individual plants, that yielded similar results. The housekeeping gene *ADP* (Kong *et al.*, 2014) was used as a standard, and its expression was uniform among the three genotypes.

The transcript levels of an SA-related gene, *Pathogenesis-related 1* (*PR1*, MELO3C023694), was elevated in the dwarf *prvΔ154* mutant, and nearly undetectable in the non-dwarf *prvΔ144* mutant and the WT. *Glutathione S-Transferase* (*GST*, MELO3C023220) transcript levels were considerably up-regulated in the dwarf *prvΔ154* mutant, compared with the non-dwarf mutant and the WT. Three other defense genes, *Cucumber pathogen-induced1* (*CuPi1*, MELO3C018878), *Putative carboxylesterase* (*CXE*, MELO3C011389), and *Galactinol synthase* (*Gol-S*, MELO3C011991), had high transcripts levels in the dwarf mutant and very low levels in the other two genotypes. In contrast to the above, transcripts levels of *Lipoxygenase* (*LOX*, MELO3C024348) were considerably lower in both mutants compared with the WT, perhaps because induction of the SA pathway repressed the antagonistic JA pathway.

*Prv* expression in the non-dwarf *prvΔ144* mutant was lower than in the WT, perhaps due to a positive feedback of active *Prv* on its own expression. On the other hand, expression of the dwarf *prvΔ154* allele was similar to that of the WT. Interestingly, the expression of the neighboring gene, *Fom-1*, was very elevated in the dwarf mutant compared with the WT allele and with *prvΔ144*. This could be the result of a general activation of defense responses, where *Fom-1* is being activated in the dwarf mutant together with many other defense-related genes (see below).

### Elevated temperature inhibits *PrvΔ154* autoimmune response

**Fig. 7.**
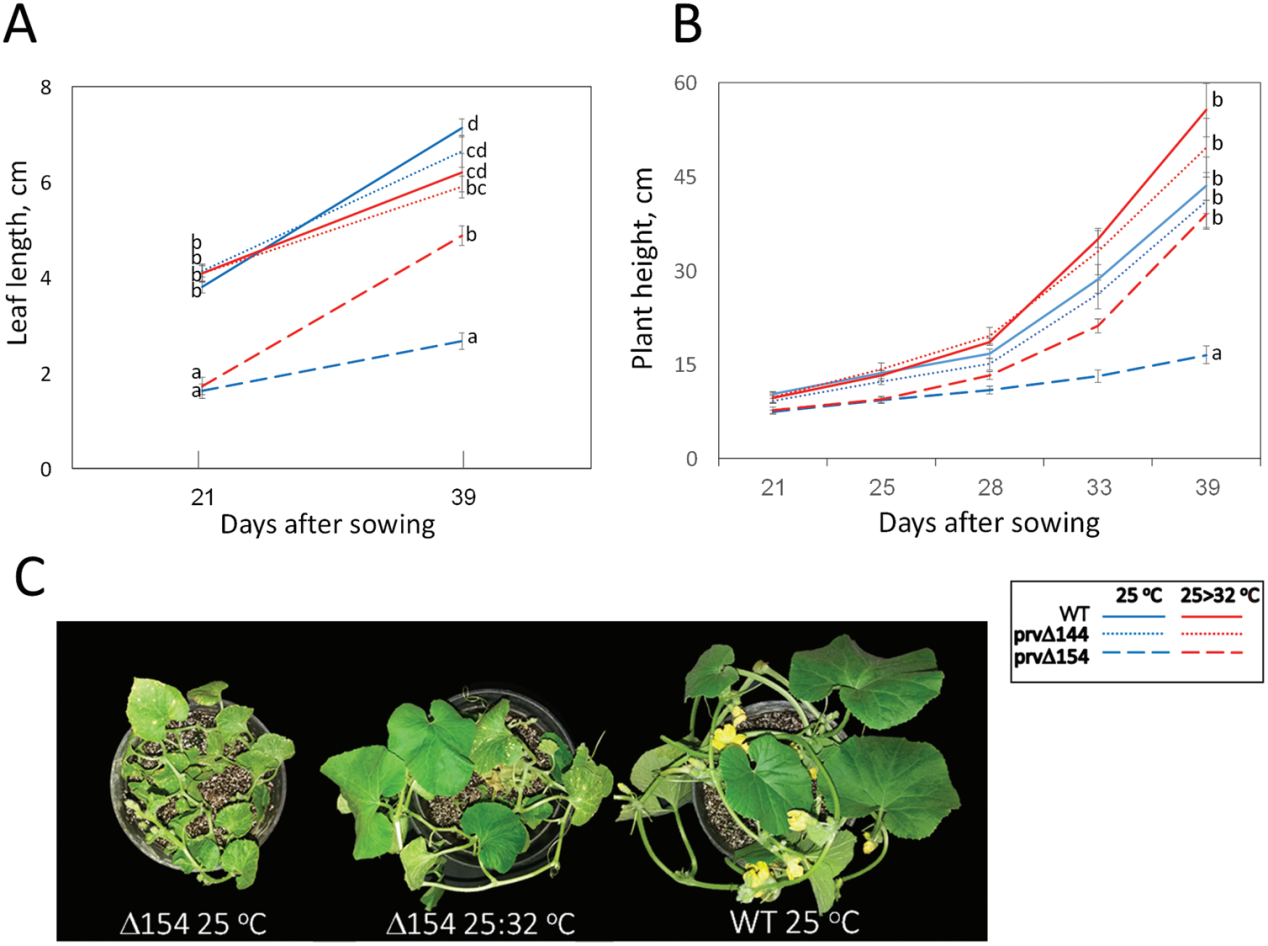
The dwarf phenotype of *prvΔ154* plants is cured by high temperature. The dwarf mutant carrying the *prvΔ154* allele was compared with WT plants (Charéntais Prv-R) and with the non-dwarf mutant carrying the *prvΔ144* allele. At 21 d after sowing, plants were exposed to two temperature regimes in the growth chamber, namely constant 25 °C and 32 °C day:25 °C night. (A) Plant height, (B) largest leaf length. Averages from at least five individual plants were analyzed by one-way ANOVA followed by Games–Howell post-hoc test; different letters indicate a significant difference at *P*<0.05. (C) Representative WT and *prvΔ154* plants, taken 1 week after transfer to the hotter regime, with the dwarf mutant already showing initial recovery, compared with the cool treatment plant that remained at 25 °C.

When we transferred several T_1_ individuals from a growth chamber with constant temperature of 25 °C to the greenhouse (~20 °C night/32–35 °C day temperatures), we found out that under the latter conditions, the dwarf *prvΔ154* plants visibly recovered within 1 week. Their newly developed stem nodes and leaves had a regular size and no lesions, becoming indistinguishable from those of WT and *prvΔ144* plants. To determine whether the phenotype of *prvΔ154* is temperature dependent, as previously reported for several autoimmune mutants (van Wersch *et al*., 2016), the growth of *prvΔ154* and *prvΔ144* mutants, as well as the WT, was monitored in growth chambers under controlled regimes. Plants were first grown at a constant temperature of 25 °C, where the extreme dwarf phenotype was expressed. At 21 d after sowing, plants were transferred to 20 °C night/32 °C day temperatures. Again, such a regime cured the autoimmune phenotype, with plant height becoming indistinguishable from that of the other two genotypes and leaf size increasing significantly (although it remained smaller than in the WT). On the other hand, plants kept at 25 °C remained dwarf. Figure 7 depicts plant height and leaf length under these conditions.

**Fig. 8.**
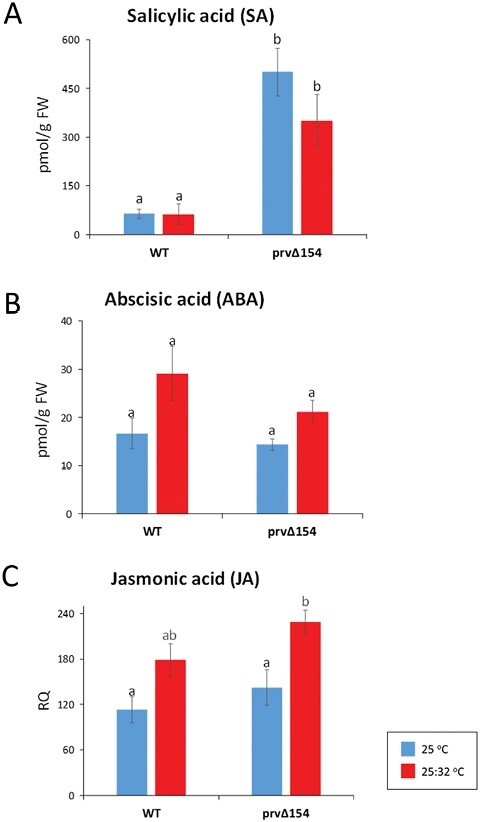
Quantification of hormones in leaf extracts from WT and *Prv* mutants under two temperature regimes. (A) SA (pmol g FW^–1^), (B) ABA (pmol g FW^–1^), (C) JA, expressed as relative quantity (RQ) among samples. Hormones were assayed in four biological replicates from separate plants using MS, as described in the Materials and methods. To compare the averages, two-way ANOVA was applied, followed by Tukey post-hoc test; different letters indicate significantly different values at *P*<0.05.

To investigate the defense response pathway activated in the mutant, SA, JA, and ABA were measured using ultra-performance liquid chromatography (UPLC) coupled with MS. Hormone levels were tested in leaf samples of WT and dwarf *prvΔ154* plants grown in growth chambers under the two temperature regimes described above. Samples were taken after 1 week of treatment. At 25 °C, SA levels in *prvΔ154* mutant leaves were ~8-fold higher than those of the WT (*P*=0.0002), showing that the autoimmune mutation is associated with activation of the SA pathway (Fig. 8A). One week after transfer to the hot regime (32 °C day/20 °C night), when the dwarf and leaf lesion phenotype of the mutant was visibly attenuated, and elevated defense transcripts have decreased (see below), SA levels were reduced by 30%, but the difference was not statistically significant. In the WT plants, SA levels did not change in response to the change in temperature.

JA levels did not differ significantly between the mutant and the WT. The temperature regime did not significantly affect the WT JA levels, while in the mutant, JA increased upon transfer to the hot regime, which could be related to the decrease in SA-related defense. ABA levels did not change significantly (but showed an increasing trend with temperature) in either the WT or the mutant (Fig. 8B, C).

**Fig. 9.**
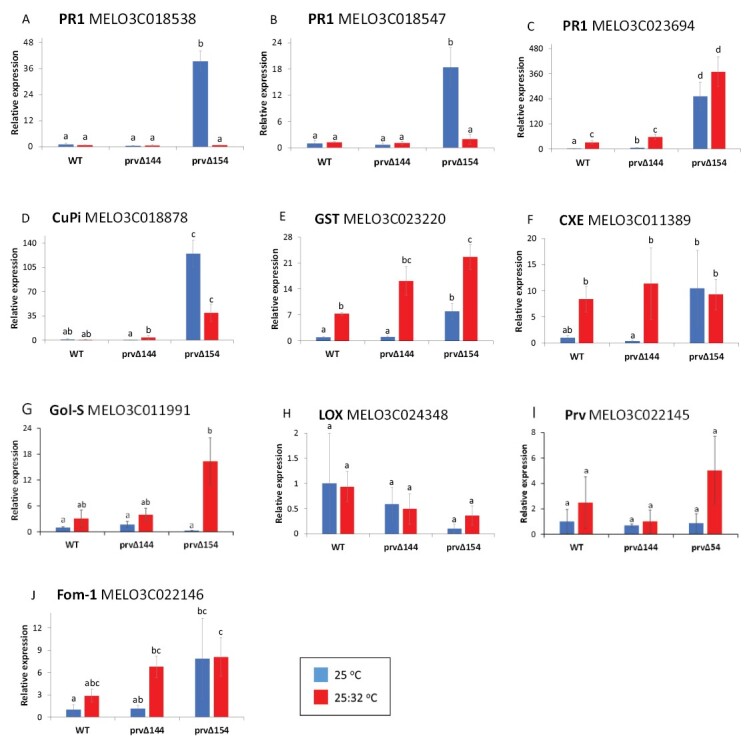
Defense gene expression in *prvΔ154* plants responds to temperature. The dwarf mutant carrying the *prvΔ154* allele was compared with WT plants (Charéntais Prv-R) and the non-dwarf mutant carrying the *prvΔ144* allele after a 1 week exposure to two temperature regimes, namely constant 25 °C and 32 °C day:25 °C night. (A–J) The transcripts of 10 defense-related genes were quantified by qRT-PCR using an automated BioMark™ HD real-time PCR System. Leaf samples included 3-4 independent biological repeats, each prepared from a single plant. RQ values were calculated with respect to the *ADP ribosylation factor 1* reference gene (MELO3C023630). Different letters indicate significant (*P*<0.05) differences between genotypes and regimes, by two-way ANOVA, followed by Tukey’s post-hoc analysis.

We wished to further confirm the temperature dependence of the elevated defense response in the dwarf mutant. We therefore assayed the expression of the genes that were selected for sqRT–PCR analysis above, as well as two additional PR1 homologs, MELO3C018538 and MELO3C018547, by qRT-PCR (genes and primers are listed in Supplementary Table S1). Leaf samples were collected from dwarf *prvΔ154*, non-dwarf *prvΔ144*, and the WT, that were grown uniformly at 25 °C and, at 21 d after sowing, were transferred for 1 week to the two alternative temperature regimes described above. Samples from different individual plants were taken in 3–4 biological replicates, RNA and cDNA were prepared, and analyzed using an automated real-time PCR system. The relative quantity of 12 defense-related genes is shown in Fig. 9.

When we compare transcript levels between mutants and WT plants at 25 °C, the qRT-PCR results broadly confirm the sqRT–PCR data, where most of the defense genes were up-regulated in the dwarf mutant, compared with the non-dwarf mutant and the WT. The effect of the high temperature regime, however, was more variable among transcripts, many of them being up-regulated in all three genotypes by the hotter temperatures. In agreement with the temperature dependence of the autoimmune phenotype, several transcripts were indeed down-regulated in dwarf plants upon transfer to the hotter regime. Three members of the PR1 family (MELO3C018538, MELO3C018547, and MELO3C023694) were significantly up-regulated (250, 40, and 20 times higher, respectively) in the dwarf *prvΔ154* plants grown at 25 °C, compared with their expression in the non-dwarf *prvΔ144* and WT plants (Fig 9A–C). When transferred to 32 °C, transcript levels of MELO3C018538 and MELO3C018547 in the dwarf mutant became very low. A similar pattern of expression was also observed for the homolog of *CuPi1* (MELO3C018878; Fig. 9D). On the other hand, MELO3C023694 transcripts were significantly up-regulated by the warmer temperature in all three genotypes, suggesting a different transcriptional control of this gene compared with the first two. *GST* (MELO3C023220) showed a similar pattern (Fig. 9E). Like most genes assayed, *CXE* (MELO3C011389; Fig. 9F) had high transcripts levels in the dwarf *prvΔ154* plants at 25 °C, compared with the non-dwarf *prvΔ144* and the WT, but at the higher temperature all genotypes showed similar levels. *Gol-S* (MELO3C011991) had similar expression patterns in the WT and the non-dwarf *prvΔ144* at both temperatures (Fig. 9G). The *prvΔ154* mutant had very low *Gol-S* expression at the lower temperatures, but upon transfer to 32 °C, it was strongly up-regulated. Also the JA biosynthesis gene, *LOX* (MELO3C024348), exhibited a contrasting expression pattern with respect to the other defense genes. *LOX* transcript levels were higher in the WT, although the differences were insignificant due to high variability among replicates. Upon transfer to warmer temperature, WT levels decreased while dwarf plant levels increased, which could reflect the well-documented antagonism between SA and JA (Fig. 9H).

*Prv* and *Fom-1* transcripts were low and variable among replicates (Fig. 9I, J), but suggested a general trend of increased expression at higher temperatures. Similar to our sqRT–PCR result (Fig. 6B), *Fom-1* transcripts were up-regulated in the dwarf *prvΔ154* mutant grown at 25 °C, but did not decrease at higher temperatures.

## Discussion

Most plant disease resistance genes (*R* genes) belong to the NLR family. NLR genes encode intracellular immune receptors, that recognize, either directly or indirectly, pathogen effectors, and initiate downstream defense responses (Takken and Tameling, 2009; van Wersch *et al*., 2020). Plant genomes contain between tens and several hundreds of NLR-homologous sequences (Ngou *et al.*, 2022), whose function, or pathogen specificity, is mostly unknown. Uncovering the function of all these sequences is a considerable challenge that depends on suitable resistant and susceptible germplasm, with reproducible disease screening methods and saturable genetic polymorphism. Moreover, after a resistance locus is mapped and a candidate *R* gene is brought forward, its function needs to be validated by reverse genetics methods, such as transgenic methods or targeted mutagenesis, to directly prove that the candidate sequence indeed controls resistance. In melon, only four *R* genes have been cloned and functionally validated so far. These include *At1* and *At2*, encoding aminotransferases that confer resistance to downy mildew, and *Vat*, an NLR gene that controls resistance to aphids, verified by overexpression in susceptible transgenic melons (Taler *et al.*, 2004; Dogimont *et al*., 2014). In addition, *mnv*, a recessive gene for resistance to melon necrotic spot virus (MNSV) encoding the translation elongation factor Cm-eIF4E, was validated using RNAi-expressing plants (Rodríguez-Hernández *et al*., 2012).

In this study, we report the functional validation of a melon TIR-NLR gene, *Prv*. The gene confers resistance to PRSV (*Potyviridae*), and it was identified by map-based cloning of the *Fom-1–Prv* locus on melon chromosome IX (Brotman *et al*., 2013). It is adjacent, in a head-to-head orientation, to the *Fom-1* gene, that confers resistance to FOM races 0 and 2. We designed a CRISPR/Cas9 knockdown vector containing two gRNAs that targeted the first exon of *Prv* (Fig. 1), and used *Agrobacterium*-mediated transformation to mutate a PRSV-resistant melon genotype, Charéntais-Prv-R. Two diploid mutated T_0_ plants were obtained, and their T_1_ progeny were tested for PRSV resistance. Both T_1_ families became fully susceptible to PRSV and expressed strong disease symptoms at 14–20 dpi. We characterized the mutations and found that Plant 1 had a large deletion (~3 kb) in both alleles, that encompassed the first exon of *Prv*, the *Fom-1/Prv* intergenic region, and the first exon of the neighboring gene, *Fom-1*. This line is therefore inactivated in both *Prv* and *Fom-1*. Mutant Plant 2 contained two different alleles with 144 bp and 154 bp deletions in *Prv*, designated *prvΔ144* and *prvΔ154*, respectively (Figs 2, 3), and we show that each of the alleles breaks PRSV resistance, because any combination of these alleles was shown to be susceptible (Fig. 5). On the other hand, plants having one WT allele and one mutated allele remained resistant to PRSV, in agreement with the well-established dominance of the *Prv*^*1*^ allele found in Charéntais Prv-R. These results validate the function of *Prv*, showing that a functional WT allele is essential for conferring resistance against PRSV infection, and represent a first report on validation of an *R* gene in melon by CRISPR/Cas9 mutagenesis.

Melon is recalcitrant to genetic transformation, having low regeneration and transformation rates (Nuñez-Palenius *et al.*, 2008). In several reports, a high percentage of the regenerated plants were tetraploid (Guis *et al.*, 2000). Tetraploid melon plants are characterized by large flowers, rounded cotyledons, short internodes, flat-shaped fruit, and round seeds (Nuñez-Palenius *et al*., 2008), as well as irregular pollen morphology (Fassuliotis and Nelson, 1992). In our study, a total of 13 independent T_0_ transgenic plants were obtained from leaf explants, consisting of two diploid and 11 tetraploid plants. The tetraploid progeny were nearly sterile and our validation was thus based on the progeny of the two diploid lines, one of which could be further separated in lines that carried two different mutated alleles. While regenerating diploid lines remains a challenge, we found that CRISPR/Cas9 rates of mutagenesis were extremely high. Upon cloning and sequencing the gRNA target regions in the mutants, we observed a 92% mutation rate, with 11 out of 13 transgenic plants harboring two mutated alleles and two plants being heterozygous, with a single mutation. Such efficiency can be attributed to successful selection of target sites, elevated expression of Cas9 under the Ubiquitin 10 promoter from *S. lycopersicum*, and the gRNAs under the U6-26 promoter. Similar results (90.4%) were observed in tomato, where the *CRTISO* gene was targeted using the same Ubi10 promoter (Dahan-Meir *et al*., 2018).

The mutations that we recovered ranged from small insertion–deletions (indels) of < 20 bp, to medium size indels (50–150 bp), up to deletions >1 kb (Table 1). The intermediate and large size deletions spanned the two *Prv* target sites. Similar results were obtained in soybean, when large fragments >4.5 kb were deleted from the *GmFT2a* gene, using a dual gRNA/Cas9 design (Cai *et al.*, 2018). The ability to delete large genomic fragments could be useful for generating null alleles and removing non-coding regions such as regulatory sequences (Wu *et al*., 2018; Tan *et al*., 2020).

The T_1_ progeny of Plant 2 showed a 3:1 segregation of a severe dwarf phenotype, with constitutive small lesions in the leaves (Figs 4, 6A). We demonstrated that one of the mutated alleles, *prvΔ154*, co-segregated with the dwarf phenotype, such that only *prvΔ154* homozygotes were dwarf, whereas plants that carried at least one *prvΔ144* or one WT *Prv* allele looked normal (Fig. 4B). Similar dwarfing mutations have been reported in several plant species, and many of them were associated with deregulation of plant defense. They were described as autoimmune mutations that constitutively express a strong defense response (van Wersch *et al*., 2016). Plant immunity is under tight negative control, and a fitness trade-off between growth and defense has been demonstrated, where defense activation slows down or even stops growth, although a few studies proposed that such a correlation could be relaxed in some cases (Karasov *et al.*, 2017). Autoimmune mutants often exhibit dwarfism, elevated SA levels, constitutive expression of defense genes, as well as enhanced disease resistance to pathogens (van Wersch *et al*., 2016). In agreement with these reports, melon plants homozygous for the *prvΔ154* allele exhibited dwarfism together with high, constitutive SA levels and high expression of several defense transcripts (Figs 6, 8, 9), confirming that *prvΔ154* is an autoimmune mutation. However, the activated defenses found in the mutant were not effective against PRSV infection. PRSV symptoms are less evident in the dwarf plants because of their stunted development, but using a GFP-expressing strain we could show that the dwarf plants are colonized by PRSV (Fig. 5C). Thus, PRSV resistance elicited by the WT *Prv* gene must rely on additional factors, other than the elevated SA and defense transcripts induced in the mutant. In future it will be interesting to see whether this mutant has become resistant to other pathogens.

In Arabidopsis, numerous reports related autoimmune phenotypes to loss-of-function mutations in negative regulators of defense, or gain-of-function mutations in plant immune receptors, such as *NLR* genes (van Wersch *et al*., 2016). We hypothesized that the *prvΔ154* phenotype results from autoimmunity and activation of downstream defense. We found that upon trypan blue staining, *prvΔ154* leaves were densely covered with stained cell clusters, suggesting that they underwent cell death (Fig. 6). Furthermore, we observed elevated expression of defense genes, such as *PR1*, in the dwarf mutant, compared with the non-dwarf *prvΔ144* mutant and the WT. This could indicate that the autoimmune phenotype is related to the SA pathway, as seen in many other *NLR* autoimmune mutants (van Wersch *et al*., 2016). Moreover, expression of a lipoxygenase transcript that belongs to the JA pathway was lower in the dwarf, in agreement with the well-established antagonism between the SA and JA pathways (Dong, 1998).

The product of the non-dwarf *prvΔ144* allele carries a 48 amino acid deletion that spans important elements of the TIR region, but the ORF is maintained. On the other hand, the *prvΔ154* dwarf mutant allele has a frameshift causing an early stop codon, resulting in a truncated protein that contains most of the TIR domain and none of the other domains (Fig. 3B). The mutated transcript still undergoes correct splicing of the first intron (Supplementary Fig. S2), and *prvΔ154* transcript levels were high (Fig. 6B), showing that nonsense-mediated decay (NMD; Shaul, 2015) has not been deployed to destroy the abnormal transcript. Interestingly, it has been suggested that during response to pathogens, plants repress NMD, because the levels of many defense transcripts are kept low by NMD (Staiger *et al*., 2013). Thus, the elevated expression of the truncated *prv* transcript could be another manifestation of defense autoimmunity.

The TIR domain of NLR proteins functions in defense signaling, and it is kept inactive by intramolecular interactions with the other domains. In many cases, overexpression of an isolated TIR domain resulted in a HR in the absence of the pathogen (Frost *et al.*, 2004; Weaver *et al.*, 2006; Swiderski *et al.*, 2009). Recently, TIR domains were found to possess NAD^+^ cleaving activity, that induces cell death in animal and plant cells (Horsefield *et al.*, 2019; Wan *et al.*, 2019), perhaps by generating a nicotinamide product that acts as a signal. It will be interesting to study the mechanism by which the *prvΔ154* allele exerts its effect; the fact that the autoimmune phenotype is recessive is less compatible with a simple ‘dominant negative’ model, where the truncated protein is constitutively signaling.

In the present study, the autoimmune symptoms caused by the *prvΔ154* allele at 25 °C were completely cured at a higher temperature (32 °C). Previous studies showed that the expression of autoimmune symptoms often depends on ambient conditions (van Wersch *et al*., 2016). For example, in Arabidopsis, a mutant allele, *chs1-2*, of the TN-type gene *CHILLING SENSITIVE 1* (*CHS1*), showed an extreme dwarf phenotype when grown at 16 °C but was cured at 22 °C (Zhang *et al.*, 2017). We note that in both Arabidopsis and melon, the higher temperatures were within the species optimal range, closer to its upper limit, but do not cause a general heat stress. At 25 °C, the dwarfed *prv* mutant had 8-fold higher SA levels with respect to the WT, consistent with the activation of an SA-dependent defense response in the mutant. After a week at 32 °C, the dwarf phenotype was cured and the SA level showed a decreasing trend.

We further hypothesized that the high temperatures that alleviate the autoimmune phenotype would also attenuate the elevated expression of defense genes in the mutant. We measured the expression of selected defense genes under the two temperature regimes and recorded different expression patterns. For example, in two *PR1* genes, MELO3C018538 and MELO3C018547, the elevated transcripts levels in the *prvΔ154* mutant decreased upon transfer to higher temperatures, in correlation with the cured autoimmune phenotype. In Arabidopsis, a single *PR1* gene (At2g14610) was induced by infection, insect attack, or chemical treatment (van Loon *et al*., 2006), and its transcript levels were 60-fold higher in an autoimmune mutant, *ubc13*, and decreased at higher temperature (Wang *et al*., 2019b). *Cucumber pathogen-induced1* (*CuPi1*, MELO3C018878), a phloem lectin gene expressed during systemic acquired resistance, was higher in the *prvΔ154* mutant and tended to decrease at elevated temperatures.

A different expression pattern was exhibited by another PR1 homolog, MELO3C023694, and by *GST* (MELO3C023220) as well as *CXE* (MELO3C011389). These genes had higher transcript levels in the dwarf mutant at 25 °C; however, upon transfer to 32 °C and curing of the phenotype, the levels did not decrease. In fact, in all three genotypes, expression increased with temperature. This could suggest that the responses initiated by the mutated *Prv* gene are diverse, and respond differently to temperature. Plant GSTs play roles in detoxification and protection against oxidative stress (Edwards *et al*., 2000) that could be related to pathogen infection. Pathogen-inducible *CXE* genes could play a role in the detoxification of pathogen-derived compounds (Ko *et al*., 2016). The melon *CXE* gene (MELO3C011389) encodes a phloem protein that accumulated upon MNSV infection, and was considered as a negative regulator of cell death (Serra-Soriano *et al.*, 2015).

In contrast to the above genes, *Gol-S* (MELO3C011991) had similar transcript levels in all genotypes at 25 °C. In fact, *prvΔ154* showed lower *Gol-S* levels but the difference was statistically insignificant. However, upon transfer to 32 °C, *prvΔ154* transcripts increased significantly. Such a pattern is a mirror image of the above-mentioned SA-related transcripts, and could result from antagonistic control, perhaps by JA. A similar trend (although not statistically significant) is also displayed by the *LOX* gene, that encodes a JA pathway enzyme (Fig. 9H). In poplar, overexpression of Arabidopsis *AtGolS* elevated galactinol content, and attenuated SA defense signaling (La Mantia *et al*., 2017).

There are numerous reports on the effect of temperature on plant immunity (Vu *et al*., 2019). For example, elevated temperatures increased susceptibility toward *Pseudomonas syringae* infection in Arabidopsis and other plant species (Cheng e*t al*., 2013). Elevated temperatures could disrupt protein interactions underlying the autoimmune response signaling; they could also destabilize the abnormal protein and reduce its activity. Moreover, accumulation and biosynthesis of SA is suppressed at high temperatures, while lower temperatures increase SA synthesis and signaling and enhance plant immunity (Huot *et al*., 2017). At optimal growth temperatures, pathogen infection will repress plant growth in favor of defense, while high temperatures reduce immunity and promote growth (Alcázar and Parker, 2011). We note, however, that WT plants that carry a functional *Prv* allele display effective resistance at 32 °C and even at 40 °C (Supplementary Fig. S3), that are frequently experienced in melon-growing regions. It appears that in our case, the autoimmune response of the dwarf mutant is qualitatively different from the WT response to PRSV controlled by *Prv*, as was already suggested by the fact that the dwarf mutant is susceptible to PRSV infection.

The *Fom-1*/*Prv* locus was located in a cluster of eight resistance gene homologs (RGHs) on melon chromosome 9. *Fom-1* and *Prv*, that confer resistance to FOM races 0 and 2, and to PRSV, respectively, encode a pair of *NLR* genes, MELO3C022146 and MELO3C022145, transcribed in opposite orientations, with 1.3 kb separating the two ORFs (Brotman *et al*., 2013). In a few well-studied cases, head-to-head oriented NLR pairs were shown to function cooperatively in immune signaling. The first NLR, known as the sensor, carries an integrated domain, which acts as a decoy for pathogen effectors, whereas the second NLR, the ‘executor’, acts as a signal transducer for defense activation. Paired NLR proteins physically interact and, in the absence of the pathogen, the sensor NLR inhibits the executor. Upon effector binding, suppression is relaxed (Cesari *et al*., 2014a; Williams *et al*., 2014; Huh *et al*., 2017). The possible physical and functional interaction between Fom-1 and Prv has not been proven yet. The fact that *Prv* contains a non-canonical domain could support the above model and designate *Prv* as the sensor and *Fom-1* as the executor. In two well-characterized R gene pairs, the executors rice RGA4 and Arabidopsis RPS4, respectively, trigger cell death in the absence of pathogen elicitation when expressed ectopically, without their cognate partner. These two ‘naturally autoactive’ NLRs are repressed by the sensor NLR of the pair, RGA5 or RRS1, respectively (Cesari *et al*., 2014b; Williams *et al*., 2014). Loss of the sensor NLR may lead to autoactivation of the executor. In rice, large-scale CRISPR/Cas9 mutagenesis of NLR pairs was performed. In agreement with the above model, most of the sensor NLR knockout mutants displayed autoimmune phenotypes. In contrast, knocking out the presumed executors only caused slight phenotypic changes (Wang *et al*., 2019a). In our case, if Prv and Fom-1 interact according to the integrated decoy model, we may speculate that the truncated *prvΔ154* protein product lost its ability to inhibit Fom-1, with the latter becoming autoactive. In the homozygous state, this could underlie the severe autoimmune phenotype, while in the heterozygous state the product of the other allele, either WT or *prvΔ144*, can still inhibit Fom-1. The product of the *prvΔ144* allele lacks essential amino acids required for responding to PRSV, but its product could still inhibit Fom-1. Future studies will focus on the Fom-1–Prv protein interaction using both the WT and mutated alleles, to test these hypotheses.

In conclusion, this study successfully applied the CRISPR/Cas9 method to validate a resistance gene in melon. The stably transformed plants underwent very high rates of mutagenesis, and the deletion alleles that were generated in a resistant genotype background broke PRSV resistance, proving the function of *Prv* in conferring resistance. In addition, one of the alleles encoded a truncated ORF that induced a dwarfing phenotype with enhanced SA-related defense. In the future we will attempt to similarly validate the adjacent *Fom-1* gene and will address the possible relationship between the two *R* genes in this locus.

## Supplementary data

The following supplementary data are available at *JXB* online.

Table S1. Primers used for this study.

Table S2. Full details of the mass spectrometric separation protocol for plant hormones.

Fig. S1. Tetraploid- versus diploid-like morphology of transgenic melons.

Fig. S2. cDNA sequence alignment of wild-type *Prv* and deletion alleles *prvΔ144* and *prvΔ154.*

Fig. S3. Response of melon genotypes Charéntais-T (PRSV susceptible) and Charéntais-PRSV-R (PRSV resistant) to PRSV inoculation at higher temperatures.

erad156_suppl_Supplementary_Figures_S1-S3_Tables_S1-S2

## Data Availability

All data supporting the findings of this study are available within the paper and within its supplementary data published online.

## Abbreviations

ABA: abscisic acid
AVR: avirulence
CRISPR/Cas9: clustered regularly interspaced short palindromic repeats/CRISPR-associated protein 9
DDW: double-distilled water
GB: Golden Braid
gRNA: guide RNA
HR: hypersensitive response
NLR: nucleotide binding-leucine-rich repeat
NMD: nonsense-mediated decay
PAMP: pathogen-associated molecular pattern
PRSV: papaya ring spot virus
qRT-PCR: quantitative real-time PCR
*R* gene: resistance gene
SA: salicylic acid
sqRT–PCR: semi-quanitative reverse transcription–PCR
TIR: Toll/Interleukin-1 Receptor
WT: wild type
