# Supplementary material for: Mutagenesis of the melon Prv gene by CRISPR/Cas9 breaks papaya ringspot virus resistance and generates an autoimmune allele with constitutive defense responses
Source: J Exp Bot. 2023 May 3;74(15):4579–96. doi: 10.1093/jxb/erad156 (PMC10433930; doi:10.1093/jxb/erad156)
Supplement: erad156_suppl_Supplementary_Figures_S1-S3_Tables_S1-S2 [file erad156_suppl_supplementary_figures_s1-s3_tables_s1-s2.pdf]

Supplementary Materials

Mutagenesis of the melon *Prv* gene by CRISPR/Cas9 breaks PRSV resistance and generates an autoimmune allele with constitutive defense responses

Shahar Nizan[1], Arieh Amitzur[1], Tal Dahan-Meir[2], Jennifer I.C. Benichou[1], Amalia Bar-Ziv[1], Rafael Perl-Treves[1]

[1]The Mina and Everard Goodman Faculty of Life Sciences, Bar Ilan University, Israel

[2]Plant and Environmental Sciences, Weizmann Institute of Science, Israel

Table S1. Primers used in this study. Pairs of primers were used for amplifying the *Npt*II and *Cas9* transgenes, the *Prv* target region, the putative off-target region, and for quantitative RT-PCR analysis of defense genes and housekeeping standards. The primer sequences are given from 5' to 3', with reference to the sequence source, as well as the amplicon size. Annealing temperatures were ~60 ^{o}C for all PCR reactions.

Purpose	Primer name	Sequence Source	Primer sequence	Product size (bp)
Transgenic analysis	nptII-F<br>nptII-R	pBINplus	AGAAGAACTCGTCAAGAAGGCGA<br>CGCCCGGTTCTTTTTGTCAAGAC	664
Transgenic analysis	Cas9-F<br>Cas9-R	*Streptococcus pyogenes Cas9*	ATGCCACAGGTGAACATCGT<br>ATCGAGGTTTGCATCAGCGA	600
Mutation analysis	*Prv* gRNA-F<br>*Prv* gRNA-R	MELO3C022145	TCACTTCCATTGTCGTTCTAAC<br>CAACTCACACTTGGATGATTTAG	607
Mutation analysis	*Prv* 200bp-F<br>*Prv* 200bp-R	MELO3C022145	CTTTGTTTTACCGGCATTTGGT<br>GCAAAATTCTGACATAGCCAAGC	206
qRT-PCR standard	ADP-F<br>ADP-R	MELO3C023630	ATATTGCCAACAAGGCGTAGA<br>TGCCCGTAAACAAGGGATAAA	93
qRT-PCR standard	RPS15-F<br>RPS15-F	MELO3C006471	GAAGCTGCGTAAAGCGAAAC<br>GGTCTTTCCATTGTAAACTCCAA	130
qRT-PCR standard	Ubi-F<br>Ubi-R	MELO3C019589	TGTTTCTAAGGTGCTGTTGTCC<br>CGTGCTGTTGCTTCATACTTG	121
qRT-PCR standard	L2-F<br>L2-R	MELO3C000111	AAACTTCTACCCCGAGCACA<br>TATGACCTCCCCCTCTATGC	150
qRT-PCR	Prv-F<br>Prv-R	MELO3C022145	CACGAAAAAATTCCAATACAATGTTC<br>GCATTTCCATTGTTCTTAATCGAAG	138
qRT-PCR	Fom1-F<br>Fom1-R	MELO3C022146	GCTTACACGAAGAACTTCTACGA<br>CCTCAACGCAATCCTTTGTTAC	138
qRT-PCR	PR1-1-F<br>PR1-1-R	MELO3C023694	GAAGAACTAAGATTTGGCCTCCTGG<br>GTTGGGATCCATTATTTTGAAGTCCT<br>C	122
qRT-PCR	PR1-2-F<br>PR1-2-R	MELO3C018538	CATGCTTCCCTCTTCTCTCGC<br>TTTGCCACCTTCTCGTCCC	114
qRT-PCR	PR1-3-F<br>PR1-3-R	MELO3C018547	CAAAACTCCCACCAAGACTTCG<br>TTGCATCTCACAAGTGCCAATCT	141
qRT-PCR	Cu-Pi1-F<br>Cu-Pi1-R	MELO3C018878	GAAGATGGTCCAAAAGAATGAACAG<br>AG<br>GGCTTATTTCTAGGGATGCAATCG	135
qRT-PCR	GST-F<br>GST-R	MELO3C023220	CGTTGAAGCTCTACTCATTCTGGG<br>GCTTTAGATACTCAGGAGCCAAATGC	138
qRT-PCR	CXE-F<br>CXE-R	MELO3C011389	GTTTCCGGTTGGCTCAGACTC<br>CGTGAGGCGGAACAGACTC	138
qRT-PCR	Gol-S-F<br>Gol-S-R	MELO3C011991	GAAGGCTCAAGGTTGCATTATTCG<br>GTACTCCACAAAGTTCCAAATTCGAA<br>G	127
qRT-PCR	LOX-F<br>LOX-R	MELO3C024348	CGAGATTGACCCAAAAACAAACGC<br>CTCTCCAAAGTCCGAGGTCAG	136
Off target analysis	gRNA2-1F<br>gRNA2-1R	MELO3C004339	GAACAACCTACTCCAGGACATGT<br>GGAACTGTCTTGACTCCCAAATTTG	254

Table S2. Mass spectrometry parameters used to detect and quantify plant hormones. MRM – multiple reaction monitoring, V – volts, eV – electron volt, CE – collision energy, IS – internal standard.

Compound	Abbrev.	Ionization mode	Cone (V)	MRM (1)	CE 1st MRM (eV)	MRM(2)	CE 2nd MRM (eV)	IS Supplier
Salicylic acid	SA	-	25	137.1 > 93	15	137.1 > 65	25	Sigma
2H4-Salicylic acid	2H4-SA	-	25	141.1 > 97	15	141.1 > 69	25	OlChemim
Abscisic acid	ABA	+	28	247.2 > 187.2	15	247.2 > 173.2	17	OlChemim
2H6-Abscisic acid	2H6-ABA	+	28	253.2 > 179.2	17	253.2 > 193.2	15	OlChemim
Jasmonic acid	JA	+	21	211.2 > 151.1	13	211.2 > 133.1	16	OlChemim
cis-12-oxo-phytodienoic acid	cisOPDA	-	30	291.2 > 165.2	20	291.2 > 247.2	20	OlChemim

Supplementary Figure S1

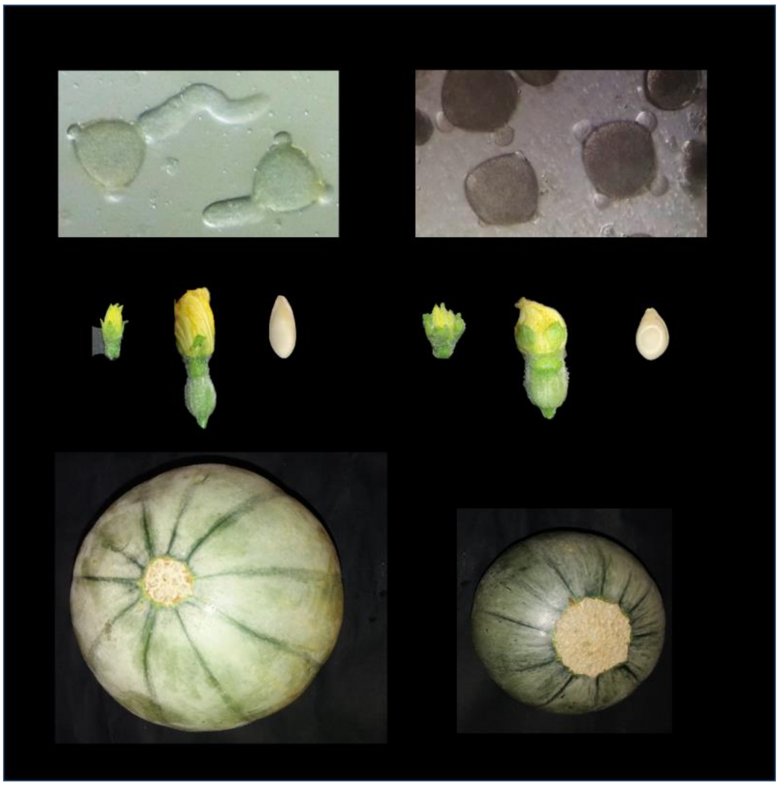

Figure S1. Tetraploid *versus* diploid-like morphology of T_0 generation melons. Left: plants that looked diploid and were fertile. Right: plants that exhibited tetraploid morphology (described by Nuñez-Palenius *et al.*, 2008) and were near-sterile. From top: pollen germination *in vitro*, male and female flowers, seeds and mature fruit.

Supplementary Figure S2

```
WT              ATGGCTGCAGGTTCCTCATCTCATCCTTCTCAAATGGCTTTTGATGTTTTCTTAAGTTTC    60
prvΔ144         ATGGCTGCAGGTTCCTCATCTCATCCTTCTCAAATGGCTTTTGATGTTTTCTTAAGTTTC    60
prvΔ154         ATGGCTGCAGGTTCCTCATCTCATCCTTCTCAAATGGCTTTTGATGTTTTCTTAAGTTTC    60
                ************************************************************

WT              AACAGAGACGAGGAAGATGATGGCTACCGACGTTTCATTAAGTGTTTATATGAGACTCTT    120
prvΔ144         AACAGAGACGAGGAAGATGATGGCTACCGACGTTTCATTAAGTGTTTATATGAGACTCTT    120
prvΔ154         AACAGAGACGAGGAAGATGATGGCTACCGACGTTTCATTAAGTGTTTATATGAGACTCTT    120
                ************************************************************

WT              AGTGAATGGGGAATCAAGATGTTTATGGATGATGATAAGAAGATGTTTATGGATGATATT    180
prvΔ144         AGTGAATGGGGAATCAAGATGTTTATGGATGATGATAAGAAGATGTTTATGGATGATATT    180
prvΔ154         AGTGAATGGGGAATCAAGATGTTTATGGATGATGATAAGAAGATGTTTATGGATGATATT    180
                ************************************************************

WT              AAGATGCTTAAGGATGATGGTAAGAAGATGTTTATGGATGACGAGGTTAATCTGAGTGAT    240
prvΔ144         AAGATGCTTAAGGATGATGGTAAGAAGATGTTTATGGATGACGAGGTTAATCTGAGTGAT    240
prvΔ154         AAGATGCTTAAGGATGATGGTAAGAAGATGTTTATGGATGACGAGGTTAATCTGAGTGAT    240
                ************************************************************

WT              GATATTGTGAAAGCAATTGAAGGATCAATCACTTCCATTGTCGTTCTAACAAAGGGGTAT    300
prvΔ144         GATATTGTGAAAGCAATTGAAGGATCAATCACTTCCATTGTCGTTCTAACAAAGGGGTAT    300
prvΔ154         GATATTGTGAAAGCAATTGAAGGATCAATCACTTCCATTGTCGTTCTAACAAAGGGGTAT    300
                ************************************************************

WT              GCTTCTTCCAAGTGGTGTTTGAGAGAGTTGGTTAAGATAATAGATCAAAAAAACAAAACC    360
prvΔ144         GCTTCTTCCAAGTGGTGTTTGAGAGAGTTGGTTAAGATAATAGATCAAAAAAACAAAACC    360
prvΔ154         GCTTCTTCCAAGTGGTGTTTGAGAGAGTTGGTTAAGATAATAGATCAAAAAAACAAAACC    360
                ************************************************************

WT              AAACACCAAGTCCTTCCTTTGTTTTACCGGCATTTGGTCCATCACGTTTATCATCAAAGT    420
prvΔ144         AAACACCAAGTCCTTCCTTTGTTTTACCGGCATTTGGTCCATCA----------------    404
prvΔ154         AAACACCAAGTCCTTCCTTTGTTTTACCGGCATTTGGTCCATCA----------------    404
                ********************************************

WT              CAAGATGTTGTTCGGCATCAAAGTCAAGATGTTGTTCAGCATCAAAGTCAAGATGTTGTT    480
prvΔ144         ------------------------------------------------------------    404
prvΔ154         ------------------------------------------------------------    404


WT              CGGCGTCAAAGTCAAGATGTTCGAAAATTCTTTAGAAGTATGGCAGAAAAAGATTACTCG    540
prvΔ144         ------------------------------------------------------------    404
prvΔ154         ------------------------------------------------------------    404


WT              GAGGTCAACTACAGGACAAGCTTGGCTATGTCAGAATTTTGCCGTCTCCCTGGAATATAT    600
prvΔ144         --------CTACAGGACAAGCTTGGCTATGTCAGAATTTTGCCGTCTCCCTGGAATATAT    456
prvΔ154         ------------------AGCTTGGCTATGTCAGAATTTTGCCGTCTCCCTGGAATATAT    446
                                  ******************************************

WT              ATATCACGAAAAAATTCcaatacaatgttccaaatcaaatccaccgcaaagcagataatt    660
prvΔ144         ATATCACGAAAAAATTCcaatacaatgttccaaatcaaatccaccgcaaagcagataatt    516
prvΔ154         ATATCACGAAAAAATTCcaatacaatgttccaaatcaaatccaccgcaaagcagataatt    506
                ************************************************************

WT              gatcgcttgcttagtcttaagctcgaagccaaagaagggaatttatttgaaatgccactt    720
prvΔ144         gatcgcttgcttagtcttaagctcgaagccaaagaagggaatttatttgaaatgccactt    576
prvΔ154         gatcgcttgcttagtcttaagctcgaagccaaagaagggaatttatttgaaatgccactt    566
                ************************************************************

WT              cgattaagaacaatggaaatgctccttggtttaggctcaaatgacatacgtttcataggg    780
prvΔ144         cgattaagaacaatggaaatgctccttggtttaggctcaaatgacatacgtttcataggg    636
prvΔ154         cgattaagaacaatggaaatgctccttggtttaggctcaaatgacatacgtttcataggg    626
                ************************************************************

WT              atagtagggatgagtggtattggtaaaacaaccccttgcggaagtgatatatgcacatagt    840
prvΔ144         atagtagggatgagtggtattggtaaaacaaccccttgcggaagtgatatatgcacatagt    696
prvΔ154         atagtagggatgagtggtattggtaaaacaaccccttgcggaagtgatatatgcacatagt    686
                ************************************************************
```

Figure S2. Alignment of cDNA sequences around the *Prv* target sites of wild type and two deletion alleles, prvΔ144 and prvΔ154. Upper-case letters - exon 1, lower-case letters - exon 2, confirming correct

splicing of the first intron. ATG start codon, and TAA stop codon in mutant Δ154 are highlighted in green and red, respectively. The two target sites are marked in yellow with the PAM motif in magenta.

Supplementary Figure S3

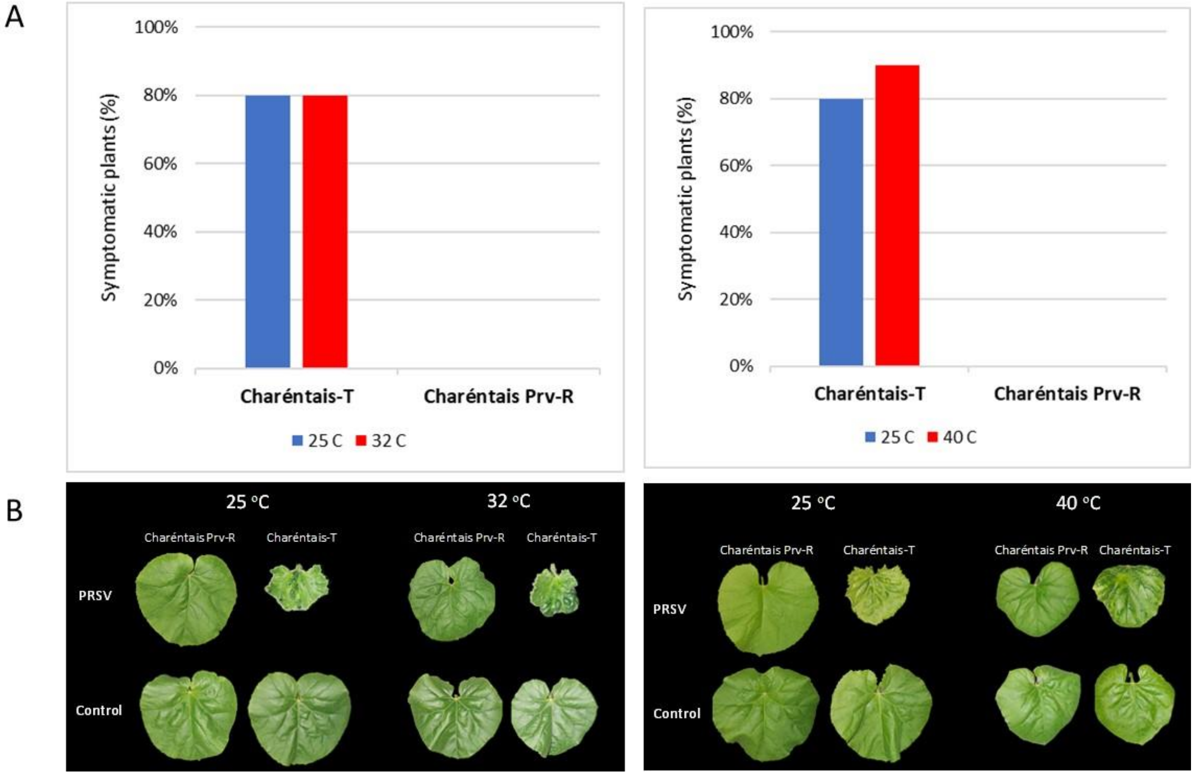

Figure S3. Response of melons to PRSV inoculation at high temperatures. Melons of the Charéntais-T (PRSV susceptible) and Charéntais-PRSV-R (PRSV resistant) near isogenic lines were germinated at 25 °C, PRSV inoculated and transferred to 32 °C day: 27 °C night (**Left** graph) or 36-40 °C day: 30 °C night temperatures (**Right**), compared, respectively, to plants kept at constant 25 °C. Ten plants of each genotype x treatment combination were scored. **A.** Percentage of plants displaying leaf mosaic and deformation at each temperature regime. Susceptible plants were highly symptomatic (80-90% disease rate), while resistant plants were fully resistant at all three regimes. At 40 °C, plants had slender stems and smaller leaves, and viral spread was somewhat accelerated. **B.** Representative leaves of inoculated (top) and non-inoculated control plants (below) from each treatment x genotype combination.
